# Pivoting from systems “thinking” to systems “doing” in health systems—Documenting stakeholder perspectives from Southeast Asia

**DOI:** 10.3389/fpubh.2022.910055

**Published:** 2022-08-04

**Authors:** Siddharth Srivastava, Devaki Nambiar

**Affiliations:** ^1^The George Institute for Global Health, New Delhi, India; ^2^Faculty of Medicine, University of New South Wales, Sydney, NSW, Australia; ^3^Prasanna School of Public Health, Manipal Academy of Higher Education, Manipal, India

**Keywords:** systems thinking, Southeast Asia, health systems, low and middle income countries (LMICs), health policy and systems research (HPSR)

## Abstract

Applications of systems thinking in the context of Health Policy and Systems Research have been scarce, particularly in Low- and Middle-Income Countries (LMICs). Given the urgent need for addressing implementation challenges, the WHO Alliance for Health Policy and Systems Research, in collaboration with partners across five global regions, recently initiated a global community of practice for applied systems thinking in policy and practice contexts within LMICs. Individual one on one calls were conducted with 56 researchers, practitioners & decision-makers across 9 countries in Southeast Asia to elucidate key barriers and opportunities for applying systems thinking in individual country settings. Consultations presented the potential for collaboration and co-production of knowledge across diverse stakeholders to strengthen opportunities by applying systems thinking tools in practice. While regional nuances warrant further exploration, there is a clear indication that policy documentation relevant to health systems will be instrumental in advancing a shared vision and interest in strengthening capacities for applied systems thinking in health systems across Southeast Asia.

## Introduction

For more than a decade, there has existed a broad consensus on Systems thinking (ST) offering strong potential, both as a lens and as a set of methods for strengthening health systems (1–3). In the wake of ever-widening health inequities exacerbated by an ongoing pandemic (4), conflict (5), and anthropogenic climate change (6), the case for moving away from reductionist approaches and viewing health systems as complex, adaptive systems is strong.

In recent years, a growing chorus calling for a shift in systems thinking from the current 'research-to practice' model toward an applied research paradigm has gained momentum (2, 7–9). The implementation of ST tools for overcoming complex healthcare system challenges across knowledge mobilization, workforce planning (10, 11), and neglected tropical diseases (12, 13), among others, has been promising. However, relative to the widespread endorsement of ST methodologies in disciplines dealing with complex systems [such as engineering, biology, and management (14, 15)], applications in the context of Health Policy and Systems Research (HPSR) have remained scarce, particularly in low- and middle-income countries (LMICs).

There are reasons for this. Firstly, despite the growing body of literature, resources available for supporting systems thinking implementation in the context of HPSR tend to emphasize conceptual writing with an almost exclusive focus on theoretical, as opposed to practical applications (16, 17). Moreover, policymakers often tend to receive abstract problem descriptions from systems scholars rather than tangible assistance and input on what ought to be done (18, 19).

Secondly, capacity-building initiatives for applied systems thinking are generally not calibrated well for adapting to existing relationships between internal (individual and organizational) and external (policy and socio-political environment) stakeholder groups across health systems (20). Long-term implementation of ST within various HPSR contexts requires stakeholders to have more than just knowledge of how ST tools can be applied–an understanding of who wields power over decision-making processes is an important consideration too (21).

Lastly, and perhaps most importantly, little is known about how policymakers actually engage with ST, or how the dynamics of collaboration between multisectoral stakeholder groups facilitate (or hinder) this engagement. Moreover, a lack of documented examples of applied systems thinking within HPSR contexts in LMIC settings further skews policymaker perceptions of ST being largely conceptual and irrelevant for policy implementation (22, 23).

Given the need for addressing these lacunae, the WHO Alliance for Health Policy and Systems Research (WHO AHPSR), in collaboration with partners across five global regions launched a Systems Thinking Accelerator (SYSTAC) in 2021 (24). Drawing from an outgrowth of learnings from the Systems Thinking for District Health Systems project implementation in Timor Leste, Pakistan, and Botswana, (25) SYSTAC was operationalized as a global community of practice for applied systems thinking in policy and practice contexts within LMICs.

Over the past year, for defining the initial engagement strategy and developing the project scope for SYSTAC, partner institutes conducted a series of regional consultations. The aim of these consultations was to: (1) Understand the needs of practitioners, researchers, and decision-makers for improving capacities in applied systems thinking across regions, (2) Elucidate key barriers and opportunities for applying systems thinking in specific settings, and (3) Catalog potential actors and initiatives in the region to explore collaborative cross-regional partnerships.

**Figure 1:**
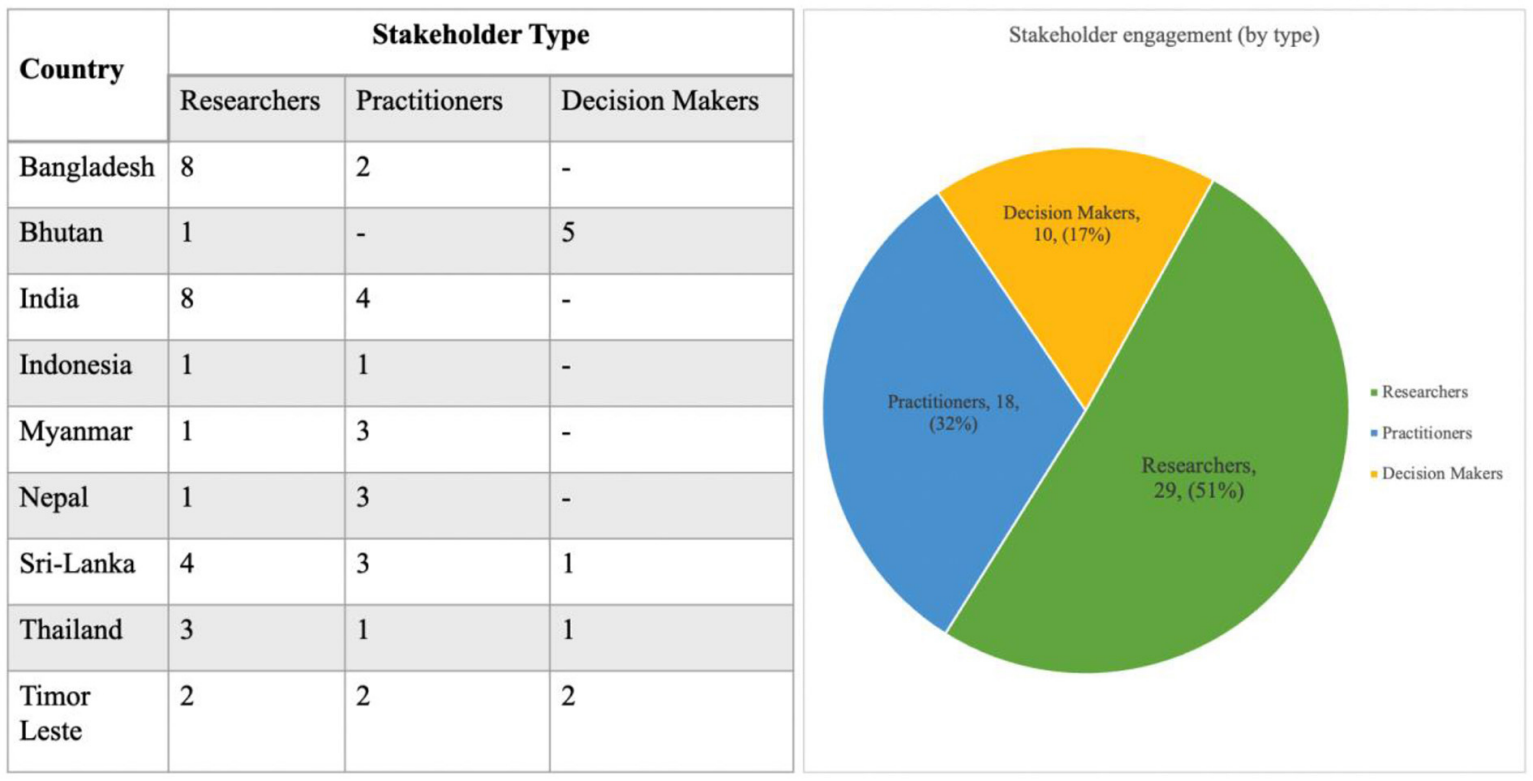
Stakeholder engagement and outreach statistics.

We present here, perspectives gathered through one-on-one virtual consultations with 56 researchers, practitioners, and decision-makers across nine Southeast Asian countries as part of a regional needs assessment, conducted between April 2021 and June 2021 (NB. outreach is still ongoing) (Figure 1). Participants were identified through existing networks, web searches of publications and institutions related to systems thinking, and recommendations of other participants and global SYSTAC network members. Virtual conversations on Zoom were held with participants ranging in duration from 30 min to over an hour and covered understandings of systems thinking, key needs, existing challenges, and future directions for driving a greater implementation of systems thinking across HPSR contexts across the region.

## The need to explore the lexicon of systems thinking in the context of Southeast Asia

Practitioners and researchers from Thailand noted that although there was an overlap between the conceptual understanding of systems thinking, approaches toward it varied across regions. In some cases, there was familiarity with and use of systems thinking, while in others there were approaches using local idioms and terminologies that could be seen to be similar to systems thinking, for example like “*Bhinneka Tunggal Ika”* (loosely translated to mean unity in diversity) in Indonesia. This was further corroborated by researchers and decision-makers in Sri Lanka, and Bhutan along with broader literature (26, 27).

While certain health reforms across the region [such as Bangladesh's constitutional commitments for social justice (28) or Bhutan's Gross National Happiness Index (29)] incorporated many of the underlying tenants of systems thinking approaches, they were not intentionally guided by the approach. Instead, these adopted an ethos of commitment to inclusion and broad-based reform, drawing upon tacit knowledge (i.e., not derived from formal research) and cultural nuances (like “*Bhinneka Tunggal Ika”* in Indonesia), which, by their nature, involved variations of classic systems thinking methodologies such as network analysis, outcome mapping, etc.

A decision-maker in Timor Leste expressed that while there was an openness to the concept of applied ST in his country, the very term “systems thinking” felt esoteric and made it daunting for broader health systems actors. A small minority of individuals also took the view that the nomenclature of systems thinking, and interrelated concepts–were all in English and predominantly adopted a western approach to implementation which could have (in part) served as an impediment to wide-scale adoption across Southeast Asia.

In the consultations, it became clear that there was a need to explore alternative regional framing similar to applied systems thinking as well as a more explicit theorization of its application in HPSR. A former deputy minister of public health in Thailand, with prior experience using ST tools, suggested introducing the concepts through the lens of “learning health systems” (30), to “explore synergies with other ongoing health systems strengthening projects across the region.”

## The need to strengthen capacities for sustained application of systems thinking in HPSR

Multiple stakeholder groups including practitioners, decision-makers, and researchers across Sri Lanka, Bhutan, Nepal, and Bangladesh expressed interest in engaging with participatory skill-building workshops demonstrating the “how” of applied systems thinking. Access to information and resources including (but not restricted to) webinars, publications, online courses, and research coalitions were identified as means to better understand the scope of applied systems thinking. Support for programming and research in the region was also called for, such that such training would not remain a disembodied, siloed exercise from ongoing regional work.

To advance this, practitioners and researchers from India suggested a potential integration of systems thinking modules into existing HPSR capacity strengthening initiatives such as the Health Innovation Fellowship (31) and the Health Policy and Systems Research fellowship (32).

For the sustainable implementation and capacity-strengthening across various contexts, however, the importance of designing a Theory of Change (ToC) (33) was underscored by multiple stakeholder groups across the region. During these discussions, an explicit emphasis was placed on considerations for delineating the scope (“how far we go”), shared understanding (“what terms we use”), and bespoke implementation strategies (“how we move things”).

## The need to demonstrate tangible, policy-relevant benefits of systems thinking approaches to implementers

Consultations with practitioners and researchers from Timor Leste, Myanmar, and Nepal (where adoption of systems thinking continues to be at a relatively nascent stage), reaffirmed that programming within ministries of health tended to default to vertical approaches for problem-solving across health systems. Such approaches, (with a narrow focus and scope) were associated with greater efficiency and higher success ratios. In these contexts, driving the adoption and implementation of systems thinking tools at a policy level continues to pose a challenge. In the absence of a priori high commitment and interest on the part of decision-makers, there was an almost unanimous regional consensus on the need to demonstrate merit in the applicability of ST methodologies in improving community health outcomes relevant to local policy contexts.

## Discussion

While a lot of the discussions during the consultations served as reaffirmations to longstanding implementation challenges of ST tools in HPSR, the findings showcase the potential for collaboration and co-production of knowledge across diverse stakeholder groups for strengthening opportunities for applying systems thinking tools in practice. Going forward, it could be interesting to study the role of collaboration in enhancing the policy-relevance of research outputs. In the context of applying ST in HPSR, understanding the value and uptake of research by policy partners, and strengthening capacities for research *via* intellectual capital (knowledge) and social capital (relationships) could be an important dimension.

The discussions also shed light on the fact that in many countries across Southeast Asia, ST may have been applied across health strengthening programs under the guise of tacit knowledge and deep-rooted cultural practices. This provides an opportunity to take note of how systems thinking is approached and practiced in different countries, which can help policymakers identify processes that could be replicated. Careful documentation of the contexts undergirding these applications and their impacts on population health outcomes is a crucial next task that must not be overlooked.

One approach for documenting these exemplars could be as part of case book compilations geared toward policymakers. Case compilations in this context could prove useful as the methodology is often recommended for presenting data in a relatively accessible manner (34, 35). Due to their focus on localized contexts, these could further assist policymakers in relating to and drawing conclusions from their own experiences. Another component for the widespread accessibility of systems thinking tools and methodologies in the context of HPSR requires a deliberate consideration of challenges posed by the unique linguistic diversity of >2,000 languages (36) across Southeast Asia. The local translation of content and resource material(s) on systems thinking could prove to be another key supplemental avenue for exploration.

Similar to the ones presented here, insights from stakeholder perspectives gathered across the global regions are being implemented across multiple, ongoing SYSTAC activities. While much remains to be explored, an overarching sentiment of fostering a shared vision and interest in strengthening capacities for applied systems thinking in HPSR across Southeast Asia is evident. Building upon this vision calls for an adherence to the heart of any systems approach–forming networks, maintaining dialogue, and actively pivoting applied systems thinking in health systems from a theory-driven (systems thinking) to an applied research (systems doing) paradigm.

## Data availability statement

The original contributions presented in the study are included in the article/supplementary material, further inquiries can be directed to the corresponding authors.

## Author contributions

DN framed, supervised the work, reviewed, and provided feedback. SS wrote the first draft and submitted the manuscript. Both authors contributed to the article and approved the submitted version.

## Funding

The authors received funding from the WHO Alliance for Health Policy and Systems Research for the needs assessments.

## Conflict of interest

The authors declare that the research was conducted in the absence of any commercial or financial relationships that could be construed as a potential conflict of interest.

## Publisher's note

All claims expressed in this article are solely those of the authors and do not necessarily represent those of their affiliated organizations, or those of the publisher, the editors and the reviewers. Any product that may be evaluated in this article, or claim that may be made by its manufacturer, is not guaranteed or endorsed by the publisher.
